# Physicians in Buffalo

**Published:** 1845-12-01

**Authors:** 


### EDITORIAL, MEDICAL INTELLIGENCE, &c.

*Physicians in Buffalo.*—The profession in other places, and especially those in the eastern states who may have in contemplation emigration westward, may like to be informed concerning the number of physicians in this city. The census which has been recently taken, enables us to give precise figures on this subject. We find on examination of the returns from the several wards, that the whole number of physicians and surgeons is 78. This is supposed to embrace medical practitioners of every denomination—Homœopathic, Botanic, Thompsonian, Uroscopic, *et id genus omne.* The number of *regular* practitioners (we cannot distinguish as *legal*, since the law now recognizes equally those of every order,) is about one half of the above number. Our population, according to the late census, is a little short of 30,000. We have thus a professed practitioner of medicine for less than every four hundred of our population. This, of course, is a great disproportion. That all cannot subsist upon the necessities of disease, is reduced to a mathematical demonstration. The ratio which the number of regular practitioners bears to the number of our population appears to be not remarkably large: but in estimating the relation between the supply and the demand, we are to take into account certain circumstances peculiar to this locality. In the first place, a large proportion, probably one fifth of our population, are foreigners, mostly Germans. This class, for the most part, employ physicians from their own country, who, with some exceptions, are not recognized in our medical associations. In addition to this, Buffalo, from its location, has a large floating, irresponsible, poor population, whose patronage contributes little or nothing toward the maintenance of the profession. To these circumstances are to be added, that our climate is healthful, and our citizens mostly young and vigorous, who have emigrated hither to place their health and activity to the best account. We have a very small proportion of the various classes of wealthy, luxurious valetudinarians, who furnish in older cities the larger portion of profitable medical business. The amount of disease compared with our population, is far less here than in any New England locality.

In view of these circumstances, Buffalo can hardly be said to offer what is technically called an "opening" for new practitioners. In this respect, however, it is not peculiar. Notwithstanding the almost magical development and growth of new towns and cities in the western world, the supply of medical practitioners every where apears to outrun the demand We have never heard from any quarter, of the scarcity of medical men; and if in the travels of those who are in pursuit of a locality where the ground is not already, in so far as numbers are concerned, amply occupied, an opening should be found, as a matter of curiosity, we should be glad to be informed of it. We have known many in search of such a place, but in every instance it has eluded discovery.

The position of Buffalo is such, that a large number of new aspirants for medical practice annually fix their locations here. Many, are disappointed, and, hence, there is, also, annually a considerable emigration from this to other places. During the last ten years probably more than the whole number of regular physicians at present established here, have moved away after a longer or shorter sojourn.

An error manifestly exists, to some extent, relative to the facilities for acquiring practice in this and other western cities. The conclusion to emigrate not infrequently involves a supposed compromise of the advantages of an older city for a more speedy attainment of an extensive practice. Experience, however, soon brings conviction of this mistake. The new candidate finds that the same obstacles, and occasions for delay exist in Western, as in Eastern cities ; and the only redeeming consideration is, that attractions of a new city, perhaps unlooked for, cause the advantages left behind to be less regretted than had been supposed, and success becomes desirable upon the same conditions. We venture also the remark, that if (as we suspect is sometimes the case) a less amount of acquirement or ability be supposod to be requisite at the West for success, it will generally prove an error which practical experience will disprove. There is probably a larger proportion of irregular and unqualified practitioners in Western than in Eastern towns, owing in a great measure, to croneous ideas to which allusion has just been made. This fact renders the medical profession, *as a profession*, perhaps less generally respected. If we mistake not, the fact *per se* of being a member of the medical profession in New England confers some little honor : at the West it confers less, and perhaps none whatever. This very fact, however, renders discrimination of individual merit and capacity more acute, and the consequence is, that those whose success implies the confidence and respect of the public, as a class, will compare with the same class in any part of the country for intelligence and acquirement.

To return to our topic—the Physicians of Buffalo—we are sure that we do not arrogate too much when we say in behalf of the fraternity to which we have the honor to belong, that we are ready at all times to extend the hand of fellowship to those who may join us, bringing proper professional credentials, and prepared to observe the rules of medical ethics, which, we are happy to add, are generally sedulously observed and cultivated by the members of the profession in this city.

